# Prediction of Sow Farrowing Onset Time Using Activity Time Series Extracted by Optical Flow Estimation

**DOI:** 10.3390/ani15070998

**Published:** 2025-03-30

**Authors:** Kejian Liu, Yigui Huang, Junbin Liu, Zujie Tan, Deqin Xiao

**Affiliations:** College of Mathematics Informatics, South China Agricultural University, Guangzhou 510642, China; 16673413675@163.com (K.L.); hyg2021scau@stu.scau.edu.cn (Y.H.); ljb@stu.scau.edu.cn (J.L.); tanzujie97@foxmail.com (Z.T.)

**Keywords:** sow, farrowing prediction, time series, optical flow estimation

## Abstract

Sow farrowing is a critical stage in pig farming, and predicting its onset can improve sow health and piglet survival. This study proposes a method based on optical flow estimation and time-series forecasting to predict sow farrowing onset. The RAFT optical flow algorithm is applied to visible-light videos of sows in late pregnancy to extract activity levels and generate time-series data. The reliability of the activity extraction algorithm is validated through correlation and trend analysis, showing strong inter-sow correlations with an average Pearson coefficient of 0.819. An in-depth analysis of sow_16 reveals a significant increase in activity 24 h before farrowing. Using these data, the CLA-PTNet model, incorporating CNN, LSTM, and attention mechanisms, is developed for continuous farrowing onset prediction. Experimental results demonstrate high predictive accuracy, with average MAE, RMSE, and R^2^ values of 5.42 min, 5.97 min, and 0.99, respectively, across four test sows. The method effectively captures activity pattern changes before farrowing, enabling precise predictions. This study offers an innovative, non-invasive solution for predicting sow farrowing onset, with significant application potential in farming practices.

## Simple Summary

Predicting the onset of sow farrowing is essential for improving sow health and piglet survival. In this study, we developed a non-invasive method to predict when sows will begin farrowing by analyzing their activity patterns from visible-light videos. By monitoring the activity levels of sows in late pregnancy, we were able to accurately predict the timing of farrowing. Our approach provides a reliable way to track sow behavior in real time, helping farmers to manage farrowing more effectively. This method has the potential to improve farm management by enabling early intervention, optimizing breeding schedules, and enhancing overall productivity in pig farming.

## 1. Introduction

Pork is one of the most consumed meats globally. According to data from the Organization for Economic Cooperation and Development (OECD), pork ranks second in global meat consumption, following poultry [1]. Global pork consumption has increased by 77%, rising from 63.5 million tons in 1990 to 113 million tons in 2022, reflecting significant growth in demand over the past decades [2]. The production and consumption of pork are not only essential for meeting human nutritional needs but also serve as indicators of socioeconomic development and dietary transitions [3,4,5]. In recent years, with the acceleration of agricultural modernization, pig farming has been transitioning toward large-scale, intelligent, and automated operations [6,7,8], gradually achieving more efficient and standardized production models. At the same time, precision farming practices that emphasize individual management and quality assurance have become the core trend in the future development of the industry [9,10,11]. The management of sows during pregnancy and farrowing is particularly critical in livestock farming. Studies have reported a dystocia rate of 6.0% at the piglet level, along with stillbirth and mummification rates of 8.7% and 1.6%, respectively; notably, nearly half (47.2%) of the farrowings experienced at least one dystocia event [12]. Therefore, proper care during late pregnancy and timely interventions during farrowing are essential to reduce the occurrence of dystocia, enhance piglet survival rates, and improve overall production efficiency [13,14,15,16]. Consequently, accurately predicting the onset of farrowing in sows has become an important research focus for optimizing management processes, enhancing economic efficiency, and ensuring animal welfare in the livestock industry [17].

The gestation length of a sow is conventionally described as 3 months, 3 weeks, and 3 days (approximately 114 days) [18,19]. However, this duration is variable, with studies reporting divergent ranges: 108–119 days [20], 111–120 days [21], and a broader span of 105–125 days [22]. Further studies have shown average gestation lengths of approximately 115.97 days, ranging from 112 to 120 days [19]; and 114.23 days, ranging from 109 to 120 days [23], highlighting the inherent variability among different pig populations. Several factors can influence gestation length, including parity, litter size, seasonal variations, and genetic background [21]. Although modern farms record insemination dates and employ production systems to estimate farrowing dates, these methods only provide approximate timeframes and cannot predict the exact timing of parturition. To enhance farrowing supervision, prostaglandins are widely administered to induce labor. This practice improves farrowing synchrony, facilitates intensive monitoring and timely interventions, and ultimately reduces piglet mortality. However, improper prostaglandin use may increase dystocia risk, highlighting the need to integrate professional supervision with medical interventions for optimal outcomes [24]. A study on cloprostenol-induced farrowings reported dystocia incidences of 11.0% at the piglet level (i.e., per individual piglet) and 75.3% at the farrowing level (i.e., per farrowing event) [25]. Therefore, developing a camera-based system for peripartum monitoring and farrowing time prediction could help reduce production costs and mitigate dystocia-related risks. Near farrowing, sows exhibit nest-building behavior, their udder becomes swollen and firm, and milk may leak from the teats [26,27,28]. While these signs help predict farrowing, the method remains labor-intensive and time-consuming, requiring constant observation by caretakers. Recent studies have explored physiological and behavioral changes in sows before farrowing, proposing various prediction methods. For instance, research has shown that sows exhibit nesting behavior and significant increases in activity levels as farrowing approaches [29,30,31]. Additionally, fluctuations in respiratory rate, surface temperature, and endocrine hormone levels have been found to be closely related to the onset of farrowing [32,33].

From a physiological perspective, studies have found that sows exhibit significant increases in respiratory rate and rectal temperature within 12 h before farrowing [34]. In parallel, researchers have employed implantable radiotelemetric temperature sensors to monitor ear base temperature in sows. This approach revealed a consistent physiological pattern: body temperature initiates a sustained rise 6–12 h before parturition [35]. Furthermore, infrared thermography has revealed that ear temperatures increase by an average of 3.4 °C within 8 h before farrowing, serving as an important predictor [17].

Regarding behavioral characteristics, researchers have used sensors to measure sow activity levels and drinking patterns, and based on the variation patterns, they have constructed hidden Markov models to predict the farrowing time of sows [36]. Additionally, some researchers have utilized photonic sensors and 3D accelerometers to measure activity levels, and through CUSUM analysis of the activity variation, they have successfully predicted the farrowing time of sows [37,38]. In other studies, accelerometer-equipped ear tags have been used to monitor sow activity changes before farrowing, employing Kalman filtering to predict sow activity. When the predicted activity exceeds a set threshold, an alarm is triggered. Although these studies have shown some predictive effectiveness, the use of invasive sensor technologies can induce stress in the sows, potentially affecting their health [39]. To overcome the limitations of invasive techniques, some researchers have employed the YOLOX object detection model to estimate sow activity levels, followed by Kalman filtering to construct a predictive model [40]. Furthermore, some researchers have developed a posture detection model for sows based on YOLOv5. By analyzing posture frequency changes from 48 h before farrowing to 24 h after farrowing, the model generates a farrowing warning when the frequency exceeds a certain threshold. This study reported an average error of 1.02 h [16].

Despite significant progress in predicting sow farrowing time, challenges remain. Most studies rely on invasive sensors for data acquisition, which not only induce stress but also limit the general applicability of the technology. On the other hand, non-invasive methods based on computer vision reduce disturbances but are often focused on specific warning times before farrowing. These methods lack the ability to perform continuous predictions of farrowing time, and prediction accuracy still needs improvement.

To address these challenges, this study proposes a continuous prediction method for sow farrowing onset based on optical flow estimation and time-series forecasting. The main contributions of this study include (1) designing an optical flow-based video analysis algorithm to extract activity features of sows during late pregnancy; (2) developing a time-series prediction model that integrates convolutional neural networks (CNNs), long short-term memory (LSTM) networks, and attention mechanisms; and (3) introducing an innovative self-attention mechanism based on learnable positional encoding and Time-Weighted adjustments.

## 2. Materials and Dataset

### 2.1. Experimental Subjects and Data Collection

The data used in this study were collected from December 2021 to July 2023 at the Danan Breeding Farm in Conghua District, Guangzhou, Guangdong Province. The experimental subjects consisted of 20 primiparous Large White sows in late pregnancy. These sows were transferred to a farrowing house approximately 10 days before their expected delivery dates. The farrowing house was equipped with 20 sow farrowing crates and featured advanced lighting, ventilation, and environmental control systems. The environmental temperature inside the house was maintained between 17 °C and 22 °C, and the humidity was controlled between 35% and 55%, providing optimal living conditions to ensure the health and comfort of the sows.

All sows were fed a standard gestation diet formulated to meet the nutritional requirements for pregnant sows, including 16% crude protein, 3200 kcal/kg digestible energy, and essential vitamins and minerals. The feeding schedule followed industry best practices, with sows provided 1.8–2.0 kg of feed per day during early gestation, 2.0–2.5 kg per day during mid-gestation, and up to 3.5–4.0 kg per day in late gestation. After farrowing, the diet was adjusted to support lactation, gradually increasing to a peak intake of 6.0 kg per day.

During the farrowing process, farm personnel regularly monitored the sows and provided assistance when necessary. If prolonged labor or difficulty in delivering piglets was observed, appropriate manual or medical interventions were performed following the farm’s standard operating procedures.

Before the trial, all sows underwent routine health checks to ensure they were free of significant illnesses or locomotor issues, such as claw lesions or lameness, which could affect their activity levels and behavior. Routine health checks were also conducted during the experimental period to monitor and maintain the health status of the sows.

**Figure 1:**
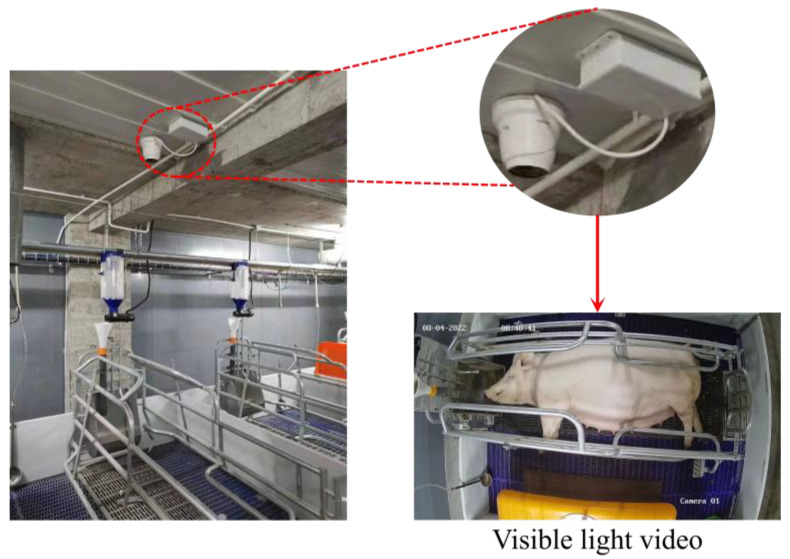
Experimental equipment setup.

To enable long-term monitoring and data collection of sow behavior, a Hikvision camera (model: TB-1217A-3/PA; Hangzhou Hikvision Digital Technology Co., Ltd., Hangzhou, China) was installed on the ceiling of the farrowing house, approximately 2.5 m above the ground. The camera was equipped with thermal-infrared and visible-light lenses and connected to a microcomputer to enable real-time video uploads to the cloud. The experimental equipment setup and the resulting video-capture quality are shown in Figure 1. The visible-light video collected had a frame rate of 25 frames per second and a resolution of 2688 × 1520, providing clear records of the sows’ behavioral characteristics.

In total, visible-light video data were collected for all 20 sows over a continuous 5-day period before and after farrowing. The sows were identified using IDs ranging from sow_01 to sow_20. This dataset provided comprehensive support for extracting and analyzing sow activity metrics and developing the farrowing time prediction model.

### 2.2. Dataset Construction

#### 2.2.1. Visible-Light Video Preprocessing

**Figure 2:**
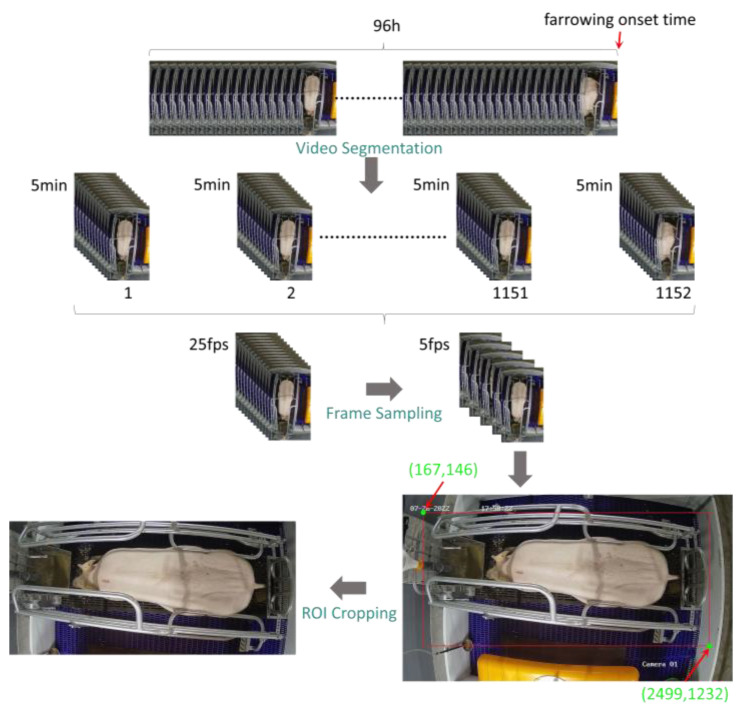
Workflow of visible-light video preprocessing.

This study uses visible-light videos as the raw data source and applies optical flow estimation algorithms to extract activity features of sows prior to farrowing. The goal is to analyze the activity patterns of sows before farrowing and provide input data for the predictive model. To ensure data quality and extract meaningful features, the raw video data undergo several preprocessing steps, including video segmentation, frame extraction, image cropping, and resolution adjustment. The detailed workflow is shown in Figure 2 and is outlined below:

(1) Video segmentation: The onset of sow farrowing (defined as the moment the first piglet is born, determined through manual review of the visible-light video) is used as the endpoint for segmentation. Starting from this point, the video is segmented into 5-min clips by moving backward in time. For each sow, a total of 1152 video clips, each 5 min long, were generated.
(2) Frame extraction: To reduce computational complexity while preserving key information, the original video frame rate of 25 frames per second (fps) was reduced to 2 fps. Specifically, the 1st and 13th frames of each second were selected.
(3) Image cropping: To focus on the activity area of the sow, each video frame was cropped to exclude irrelevant regions and highlight the region of interest (ROI). A red rectangle was first used to mark the ROI in the image, with the top-left corner coordinates at (167, 146) and the bottom-right corner coordinates at (2499, 1232), resulting in a rectangle size of 2332 pixels in width and 1086 pixels in height. Based on this marked ROI, all video frames were cropped, ensuring that the dataset retained only the activity information within the target area.

Through these preprocessing steps, the data size was significantly reduced, while the critical activity features of the sows were effectively retained. These preprocessed data lay a solid foundation for subsequent analysis of farrowing behavior patterns and the construction of the predictive model.

#### 2.2.2. Construction of Sow-Activity Time-Series Dataset

Using the optical flow estimation algorithm, activity data were extracted for 20 late-pregnancy sows (sow_01 to sow_20) and recorded in a time-series format. The data collection window was set to span the 96 h leading up to the onset of farrowing, with a sampling frequency of one data point every 5 min (corresponding to one 5-min video segment). As a result, each sow’s activity data consisted of 1152 time points within the 96-h window. To reduce the influence of outliers, the time-series data were smoothed during preprocessing.

**Figure 3:**
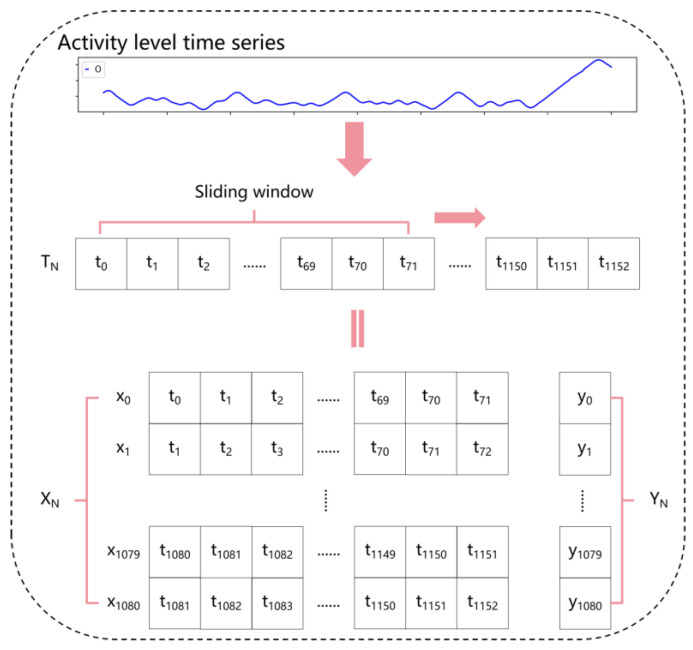
Sliding window and data labeling process.

Following this process, the initial time-series dataset was obtained. To prepare the data for input into the predictive model, a sliding window approach was applied to segment the time series, as illustrated in Figure 3. For a single sow’s time-series data, the sliding window was set to a length of 6 h (72 data points) with a step size of 1. This generated 1081 samples per sow, with each sample containing continuous data for a 6-h period. Each sample was assigned a label indicating the time difference (in minutes) between the final data point in the sample and the onset of farrowing for the respective sow. This means that the label represents the remaining time until farrowing at that specific moment in the time series, expressed in minutes.

By applying the above processing steps to the activity data of 20 sows, the final sow activity time-series dataset was constructed. As shown in Table 1, 16 sows were randomly selected for training and validation, with 4-fold cross-validation used to optimize model parameters and evaluate performance. The remaining 4 sows were used as the test set to assess the model’s performance in practical applications.

## 3. Methods

### 3.1. Overview of the Methodology

**Figure 4:**
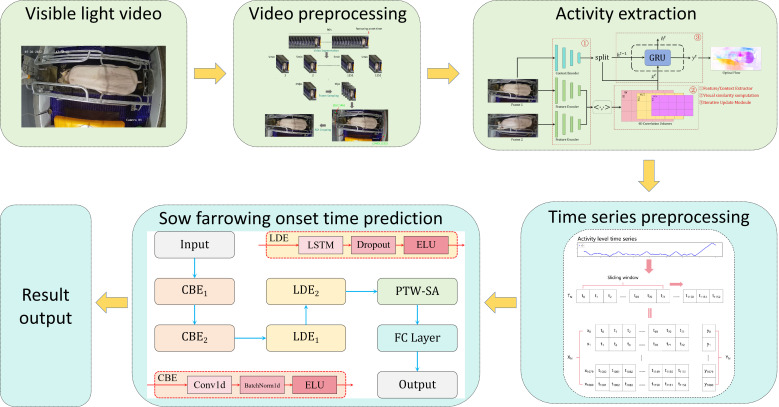
Workflow for predicting the onset of sow farrowing. Abbreviations: CBE (Convolutional Block for Feature Extraction, Conv1D + BatchNorm + ELU), LDE (Long-Term Dependency Encoder, LSTM + Dropout + ELU), PTW-SA (Position-Aware Time-Weighted Self-Attention), and FC (fully connected layer). Arrows indicate data flow between modules.

This study aims to predict the onset of sow farrowing based on optical flow estimation and time-series forecasting using visible-light video data. The overall research workflow is illustrated in Figure 4. First, visible-light video data of late-pregnancy sows are collected using cameras. The raw video data are then preprocessed to ensure quality and usability. Next, the optical flow estimation algorithm is applied to extract the activity levels of sows from the video frames, which are subsequently transformed into activity time-series data. To ensure compatibility with the input requirements of the predictive model, the time-series data undergo further preprocessing. Finally, based on the processed time-series data, a time-series prediction model is used to predict the onset of farrowing, producing the final prediction results.

### 3.2. Sow Activity Extraction Algorithm Based on Optical Flow Estimation

Optical flow estimation is a motion estimation technique widely used in the field of computer vision. It is primarily utilized to detect and estimate the motion vectors of pixels in a sequence of images or video frames. In this study, we apply an optical flow estimation algorithm to detect pixel movement between consecutive frames in sow videos, thereby quantifying the activity level of the sows.

**Figure 5:**
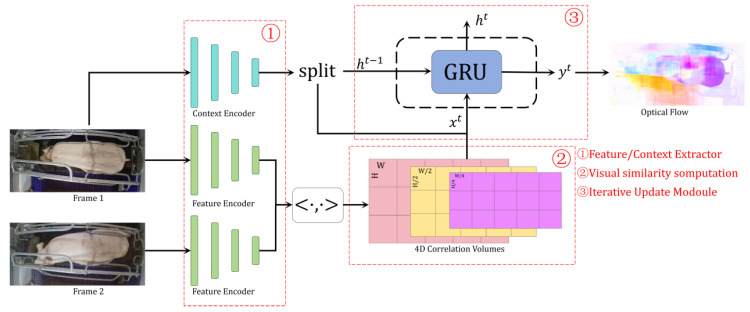
RAFT network structure.

The algorithm used in this study is RAFT (Recurrent All-Pairs Field Transforms), an end-to-end deep learning network proposed by [41]. RAFT has demonstrated excellent performance across multiple benchmark datasets, known for its computational efficiency and robustness. The RAFT network structure is illustrated in Figure 5. The network comprises three main components: the encoder, the visual similarity computation module, and the iterative update module. The encoder is further divided into the feature encoder and the context encoder. The feature encoder extracts feature vectors for each pixel in the first and second input frames, producing feature vector pairs, $g_{\theta }\left(I_{1}\right)$ and $g_{\theta }\left(I_{2}\right)$. The context encoder extracts contextual information from the first input frame. Both encoders are built using residual blocks, but the normalization methods differ: the feature encoder uses instance normalization, while the context encoder uses batch normalization. After processing by the encoders, the size of the feature map is reduced to 1/8 of the original image dimensions. The visual similarity computation module calculates the similarity between feature vector pairs, $g_{\theta }\left(I_{1}\right)$ and $g_{\theta }\left(I_{2}\right)$. The iterative update module, based on gated recurrent unit (GRU) operators, iteratively updates the optical flow estimates. The inputs to this module include the visual similarity features of $g_{\theta }\left(I_{1}\right)$ and $g_{\theta }\left(I_{2}\right)$, the initial optical flow information, and the contextual information. The iterative process mimics traditional optimization algorithms, allowing the module to refine the optical flow estimates. RAFT’s design integrates feature extraction, correlation computation, and iterative updates, enabling end-to-end optical flow estimation. The correlation computation captures rich motion information, while the iterative update module simulates the refinement process, producing highly accurate optical flow results across diverse application scenarios.

When RAFT is applied to video data, each pair of consecutive frames generates an optical flow field represented as a three-dimensional tensor, F=[H,W,2]. Here, H represents the image height; W represents the image width; and the third dimension represents a two-dimensional vector [u,v], where u denotes the horizontal motion component, and v denotes the vertical motion component. The magnitude of the displacement is determined by the numerical value, and the direction of motion is indicated by the sign.

To quantify sow activity, the average displacement magnitude of all pixels in each optical flow field is computed as follows:

(1) $$A=\frac{1}{N}\sum_{i=1}^{N}\sqrt{u_{i}^{2}+v_{i}^{2}}$$

where A represents the mean displacement magnitude per frame; N is the total number of pixels in the optical flow field; and $u_{i}$ and $v_{i}$ are the horizontal and vertical displacement components of the i-th pixel, respectively. The unit of measurement for A is pixels per frame.

Since each preprocessed video segment spans 5 min and is sampled at 2 frames per second, optical flow estimation is applied to all frames within this duration. The total activity level for a given 5-min period is then calculated as the sum of the frame-wise activity levels:

(2) $$A^{’}=\sum_{t=1}^{T}A_{t}$$

where $A^{’}$ represents the total activity level for the 5-min segment, and T denotes the total number of frames in the segment (T = 600T = 600 frames, given a frame rate of 2 fps over 5 min). The unit of $A^{’}$ remains in pixels, representing the cumulative displacement magnitude over the segment.

Since the raw displacement values are inherently large due to pixel-level calculations, normalization is required before feeding them into the predictive model. To scale the activity values into a more manageable range, min–max normalization is applied, transforming the values into a standardized range from 0 to 100:

(3) $$A^{\prime }=\frac{A^{’}-{A^{’}}_{min}}{{A^{’}}_{max}-{A^{’}}_{min}}\times 100$$

where $A^{\prime }$ is the normalized activity level; and ${A^{’}}_{min}$ and ${A^{’}}_{max}$ represent the minimum and maximum values in the dataset, respectively. This normalization enhances model stability, prevents extreme values from dominating training, and improves interpretability by making the activity metric consistent across different sows.

Additionally, a similar normalization process was applied during the analysis of sow activity levels to facilitate comparisons between different individuals and time periods. This approach reduces the influence of extreme outliers and allows for the identification of behavioral trends associated with farrowing onset.

By aggregating, normalizing, and analyzing the frame-wise optical flow information, this method effectively quantifies sow movement over time, ensuring both numerical stability for machine learning applications and interpretability in behavioral analysis.

### 3.3. Construction of the CLA-PTNet Model

#### 3.3.1. Structure of CLA-PTNet

**Figure 6:**
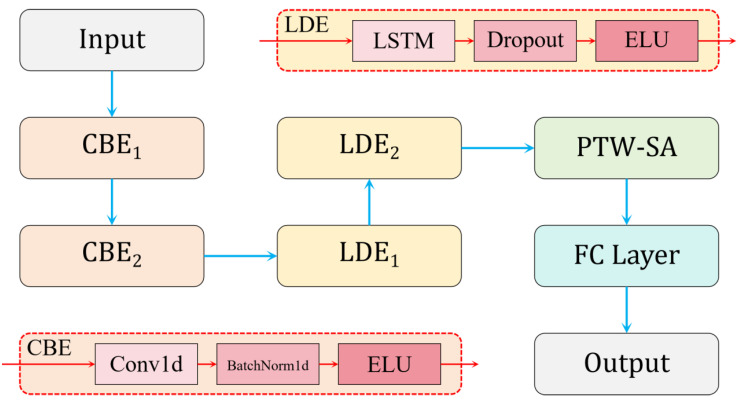
Structure of CLA-PTNet: a hybrid CNN-LSTM–Attention Architecture. Abbreviations: CBE (Convolutional Block for Feature Extraction, Conv1D + BatchNorm + ELU), LDE (Long-Term De-pendency Encoder, LSTM + Dropout + ELU), PTW-SA (Position-Aware Time-Weighted Self-Attention), and FC (fully connected layer). Arrows indicate data flow between modules.

In this study, a predictive model named CLA-PTNet was developed by integrating convolutional neural networks (CNNs), long short-term memory (LSTM) networks, and attention mechanisms. The structure of CLA-PTNet is shown in Figure 6. The model is composed of three main modules: the CBE module, the LDE module, and the PTW-SA module.

First, the preprocessed time-series data are input into the CBE module, which is designed to extract local features and multi-scale features from the time series. The CBE module utilizes one-dimensional convolutional layers (Conv1d) for feature extraction and applies batch normalization (BN) layers to normalize data within each batch. This enhances the stability of the model and accelerates its convergence during training. Additionally, the Exponential Linear Unit (ELU) activation function is employed to introduce non-linearity, thereby improving the expressive power of the network. Next, the LDE module is responsible for handling long-term dependencies within the time-series data. By employing an LSTM network, the LDE module is capable of capturing critical information in the time series and retaining key temporal relationships. To enhance the model’s generalization ability and reduce overfitting, the LDE module incorporates dropout layers. Like the CBE module, the LDE module also uses the ELU activation function for non-linear transformations. Finally, the PTW-SA (Position-Aware Time-Weighted Self-Attention) module uses an attention mechanism to assign varying weights to the outputs of the LSTM, emphasizing the important segments within the time series. By focusing on the most relevant parts of the data, the PTW-SA module enhances the model’s ability to capture meaningful patterns. The output of the PTW-SA module is then passed to a fully connected layer, which generates the final predictive output.

This modular design allows CLA-PTNet to effectively extract, process, and interpret time-series data, making it well-suited for precise predictions in complex temporal prediction tasks.

#### 3.3.2. Design of PTW-SA

LSTM networks are well known for their ability to capture long-term dependencies in time-series data. However, in practical applications, the contribution of different time steps is often uneven. Some time steps may contain key, predictive information, while others may include irrelevant data that can negatively impact the model’s performance. Traditional LSTM models do not explicitly model the relative importance of different time steps, nor do they fully consider how each feature in the time series contributes to the final prediction. This limitation makes it difficult for LSTMs to effectively focus on the time steps with the highest predictive value in some tasks.

To address this issue, this study proposes a Position-Aware Time-Weighted Self-Attention (PTW-SA) mechanism, which incorporates learnable positional encoding and Time-Weighted mechanisms. The goal of PTW-SA is to help the model focus on the most critical time steps by introducing time-step positional information and Time-Weighted attention. Specifically, the PTW-SA mechanism combines time-step positional information with weighted adjustments, allowing the model to more precisely capture key time points and temporal patterns in the sow activity sequence. This enhances prediction accuracy by enabling the model to focus on the most relevant segments of the time series. Through this design, PTW-SA overcomes the limitations of traditional LSTM models and effectively addresses the imbalance of time-step importance, improving the model’s performance in farrowing prediction tasks.

**Figure 7:**
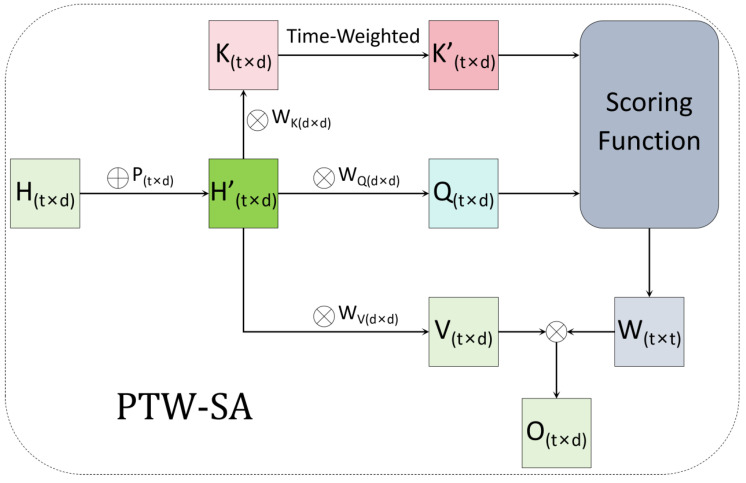
Architecture of Position-Aware Time-Weighted Self-Attention (PTW-SA) mechanism. Abbreviations: H (hidden state matrix, output of LSTM, $H\in R^{t\times d}$), $H^{’}$ (weighted sequence matrix, $H^{’}\in R^{t\times d}$), Q (query matrix), K (key matrix), V (value matrix), $K^{’}$ (weighted key matrix), W (attention weights matrix), O (output matrix). Parameters: t (time steps); d (hidden dimension); $W_{Q},W_{K},W_{V}$ (learnable projection weights). Solid arrows denote data flow.

As shown in Figure 7, the PTW-SA structure works as follows: when given time-series data, $X=\{x_{1},x_{2},\ldots ,x_{t}\}$, where $X\in R^{n}$, an LSTM network processes these data to produce the hidden state sequence matrix $H=\{h_{1},h_{2},\ldots ,h_{t}\}\in R^{t\times d}$, where t is the sequence length, and d is the dimension of the LSTM hidden layer.

To introduce time-step information, we add learnable positional encoding for each time step. Specifically, we use a trainable matrix, $P={[p}_{1},p_{2},\ldots ,p_{t}]$, where each positional encoding $p_{i}\in R^{d}$ corresponds to a learnable vector for time step i. By adding this positional encoding matrix, P, to the LSTM output matrix, H, we obtain a weighted sequence matrix, $H^{’}=H+P$, where $H^{’}\in R^{t\times d}$. This matrix contains the features of the input sequence, along with the explicit encoding of the relative positional information of each time step.

Next, to pass this information into the self-attention mechanism, we generate the query, key, and value matrices required for attention through three fully connected layers:

(4) $$Q=H^{’}W_{Q},\;K=H^{’}W_{K},\;V=H^{’}W_{V}$$

where $W_{Q},W_{K},{and\;W}_{V}\in R^{d\times d}$ are the weight matrices for the query, key, and value, respectively; and $Q,\;\;K,\;and\;V\in R^{t\times d}$ represent the resulting matrices.

We then introduce a learnable Time-Weighted weight vector, $W_{time}={\left[w_{1},w_{2},\ldots ,w_{t}\right]}^{T}\epsilon R^{t}$, where each $w_{i}$ corresponds to the Time-Weighted coefficient for the i-th row of the key matrix. We apply this Time-Weighted adjustment to each row of the key matrix, K, as follows:

(5) $$K^{’}[i,:]=w_{i}\cdot K[i,:]$$

where $K^{’}\epsilon R^{t\times d}$ is the Time-Weighted key matrix. During training, the model automatically adjusts the values of $w_{i}$ to give higher weight to time steps closer to the farrowing event, thereby enhancing the model’s focus on these crucial time points.

After applying the Time-Weighted adjustment, the weighted key matrix, $K^{’}$, is combined with the query matrix, Q, and passed into a scoring function to compute the attention scores, resulting in the attention weight matrix, W. The formula for this process is as follows:

(6) $$W=Softmax(\frac{Q{K^{’}}^{T}}{\sqrt{d}})$$

where d denotes the dimensionality of the Q and $K^{’}$ vectors. The function Softmax(·) is applied row-wise to normalize the attention scores into a probability distribution. It is defined as follows:

(7) $$Softmax(z_{i})=\frac{e^{z_{i}}}{\sum_{j=1}^{t}e^{z_{j}}},i=1,2,\ldots ,t$$

where $z_{i}$ denotes the attention score corresponding to the i-th time step, and t is the sequence length. The denominator represents the exponentiated sum over all attention scores within the sequence.

Finally, the value matrix, V, is multiplied by the attention weight matrix, W, to obtain the final output matrix, O:

(8) O=WV

In this process, the model effectively concentrates its attention on the important time steps that are closer to the farrowing event, achieving precise predictions of the farrowing time through the Time-Weighted attention mechanism. This approach allows the model to improve its predictive accuracy by focusing on the most relevant temporal information.

## 4. Results and Analysis

### 4.1. Experimental Platform

The model training and testing in this study were conducted in a Windows 10 operating system environment. The hardware used is as follows: 11th Gen Intel(R) Core (TM) i5-11400 @ 2.60 GHz, NVIDIA GeForce RTX 3070 with 24 GB VRAM, and 16 GB RAM. The software environment used for implementation includes Python 3.9, Pytorch 1.9, OpenCV 4.6.0, MMcv-full 3.17, Pandas 1.1.4, Scikit-learn 1.3.2, CUDA 11.23, and cuDNN 8.0.

### 4.2. Evaluation Metrics

To evaluate the performance of CLA-PTNet in predicting sow farrowing onset time, we used three evaluation metrics: mean absolute error (MAE), root mean squared error (RMSE), and the coefficient of determination (R^2^). MAE reflects the average deviation between the predicted values and the actual values, providing an intuitive measure of prediction accuracy. RMSE, in comparison to MAE, is more sensitive to larger errors, making it a comprehensive metric that evaluates the model’s performance across various error levels. R^2^ reflects the degree of fit between the predicted values and the actual values, providing insight into how well the model captures the data’s underlying patterns. The mathematical formulas for these metrics are as follows:

(9) $$MAE=\frac{1}{n}\sum_{i=1}^{n}\left|y_{i}-{\hat{y}}_{i}\right|$$

(10) $$RMSE=\sqrt{\frac{1}{n}\sum_{i=1}^{n}{\left(y_{i}-{\hat{y}}_{i}\right)}^{2}}$$

(11) $$R^{2}=1-\frac{\sum_{i=1}^{n}{\left(y_{i}-{\hat{y}}_{i}\right)}^{2}}{\sum_{i=1}^{n}{\left(y_{i}-\bar{y}\right)}^{2}}$$

where n represents the length of the data, $y_{i}$ represents the actual value of the ith data point, ${\hat{y}}_{i}$ represents the predicted value of the ith data point, and $\bar{y}$ represents the mean of all actual values.

In addition to these prediction evaluation metrics, we also considered End-Point Error (EPE) and the Fl-all metric to assess the performance of RAFT, which was used for sow activity extraction. Since constructing an optical flow dataset for sow activity estimation is highly challenging, we relied on publicly available benchmark datasets to evaluate RAFT’s optical flow estimation accuracy.

End-Point Error (EPE) measures the average Euclidean distance between the predicted and ground truth optical flow vectors for each pixel. It is widely used to assess optical flow estimation accuracy, with lower values indicating higher precision. The formula for EPE is as follows:

(12) $$EPE=\frac{1}{N}\sum_{i=1}^{N}\sqrt{{\left(u_{i}-{\hat{u}}_{i}\right)}^{2}+{\left(v_{i}-{\hat{v}}_{i}\right)}^{2}}$$

where N is the number of pixels, $\left(u_{i},v_{i}\right)$ are the ground-truth optical flow components, and $\left({\hat{u}}_{i},{\hat{v}}_{i}\right)$ are the predicted optical flow components.

The Fl-all metric represents the proportion of pixels where the estimated optical flow deviates significantly from the ground truth, typically when the error exceeds a predefined threshold. This metric is useful for evaluating model robustness, with lower Fl-all values indicating fewer large motion estimation errors. The formula for Fl-all is as follows:

(13) $$F1-all=\frac{\sum_{i=1}^{N}1({EPE}_{i}>\delta )}{N}\times 100\%$$

where 1(·) is an indicator function that equals 1 if the condition inside holds, and 0 otherwise; and δ is the error threshold (commonly set to 3 pixels or 5% of the optical flow magnitude).

### 4.3. Comparison of Different Optical Flow Estimation Models

To ensure the accurate extraction of sow activity from video sequences, we evaluated multiple State-of-the-Art optical flow estimation models, including FlowNet2 [42], GMA [43], MaskFlowNet [44], PWC-Net [45], and RAFT [41]. Each model was trained on the FlyingThings3D dataset and subsequently tested on three widely used benchmarks: Sintel Clean, Sintel Final, and KITTI2015. These datasets provide a comprehensive evaluation of optical flow performance under different conditions. Sintel Clean represents high-quality, noise-free synthetic data, while Sintel Final introduces realistic rendering effects, such as motion blur and lighting variations, making it more challenging. KITTI2015, on the other hand, is a real-world dataset collected from urban driving scenes, offering a test environment that involves natural lighting changes and background complexity, which is beneficial for assessing model robustness.

The experimental results presented in Table 2 demonstrate that RAFT consistently outperformed other models in terms of accuracy, robustness, and computational efficiency. RAFT achieved the lowest End-Point Error (EPE) across all three datasets, with values of 1.38 on Sintel Clean, 2.79 on Sintel Final, and 4.95 on KITTI2015. These results indicate that RAFT is particularly effective in estimating motion dynamics across both synthetic and real-world settings. Compared to alternative models such as FlowNet2 and MaskFlowNet, RAFT exhibited a substantial reduction in error rates, particularly in the more challenging KITTI2015 dataset. Furthermore, the Fl-all error, which measures the proportion of large optical flow errors, was significantly lower for RAFT, at 16.23%, whereas FlowNet2 and MaskFlowNet reported considerably higher values of 28.28% and 29.70%, respectively. This suggests that RAFT provides more precise and stable motion estimation, which is critical for tracking subtle activity variations in sows confined within farrowing crates.

Beyond its accuracy, RAFT also offers significant advantages in computational efficiency. With only 5.3 million parameters, it is considerably lightweight compared to models such as FlowNet2, which contains 162 million parameters, making the latter computationally expensive and impractical for real-time applications.

In conclusion, the comparison of different optical flow models demonstrated that RAFT achieves the best balance of accuracy, robustness, and computational efficiency, making it the optimal choice for sow activity estimation.

### 4.4. Sow Activity Data Analysis

#### 4.4.1. Correlation Analysis of Sow Activity

**Figure 8:**
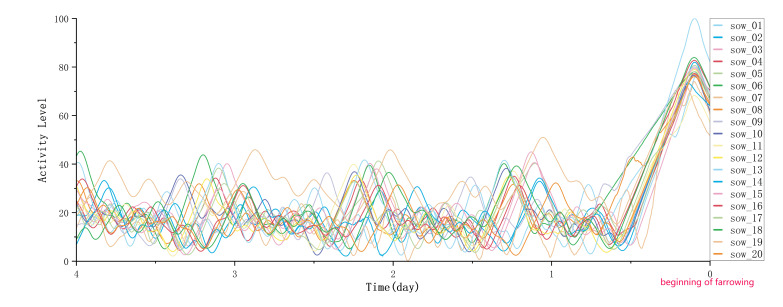
Variation in sow activity relative to farrowing onset. Time points on the x-axis: 0 (onset of farrowing), 1 (24 h before onset), 2 (48 h before onset), 3 (72 h before onset), and 4 (96 h before onset). Activity levels are normalized to a 0–100 scale after smoothing.

After extracting the sow activity time-series data using optical flow estimation, we performed smoothing and normalization (scaling the data to a range from 0 to 100) to facilitate subsequent analysis. Figure 8 shows the activity variation curve of the sows before farrowing after smoothing and normalization.

We first conducted a correlation analysis of the extracted sow activity data based on the Pearson correlation coefficient (PCC). The Pearson correlation coefficient is a metric used to measure the linear relationship between two sets of data, with values ranging from −1 to 1. The formula for calculating PCC is as follows:

(14) $$\rho_{X,Y}=\frac{\mathrm{cov}(X,Y)}{\sigma_{X}\sigma_{Y}}$$

where cov⁡(X,Y) represents the covariance of X and Y; and $\sigma_{X}$ and $\sigma_{Y}$ represent the standard deviations of X and Y, respectively.

**Figure 9:**
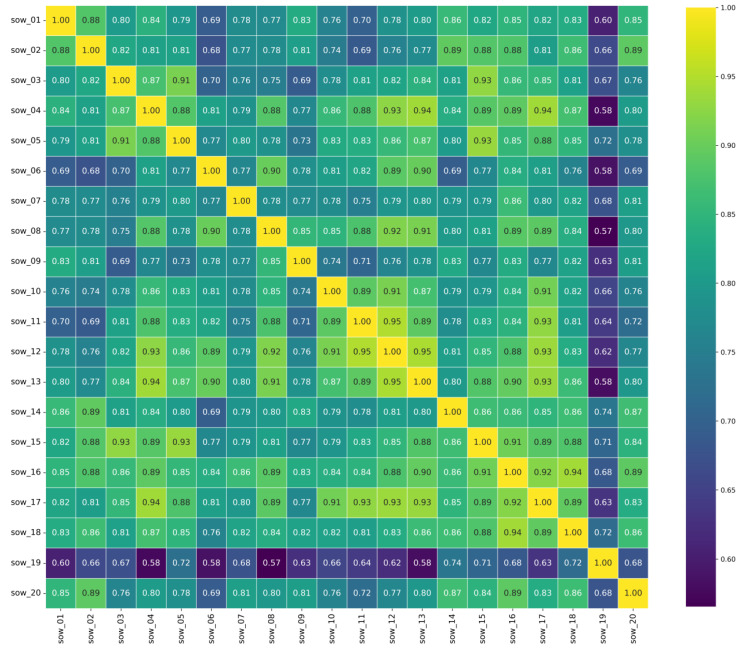
Heatmap of Pearson correlation coefficients between sow activities.

As shown in Figure 9, the minimum activity correlation coefficient between sows was 0.57, and the average correlation coefficient was 0.819, indicating a strong correlation in activity variation across all sows. Notably, for some sows, the correlation coefficient exceeded 0.9, suggesting that their activity changes were almost perfectly positively correlated. This result indirectly validates the reliability of the optical flow estimation method used to extract sow activity from video.

As recorded in Table 3, the average activity correlation coefficient (AACC) between each sow and the other 19 sows was calculated. Specifically, the calculation formula for AACC is expressed as follows:

(15) $${AACC}_{i}=\frac{1}{N}\sum_{j=1}^{N}\rho_{i,j}$$

where ${AACC}_{i}$ denotes the average activity correlation coefficient for the i-th sow, N represents the total number of sows included in the study, and $\rho_{i,j}$ is the Pearson correlation coefficient (PCC) between the activity time series of sow i and sow j.

The highest AACC was 0.870 (for sow_16), indicating that the activity pattern of sow_16 was most strongly correlated with the activity patterns of other sows. Therefore, sow_16 was selected as the representative for further analysis of sow activity variation patterns.

#### 4.4.2. Analysis of Sow Activity Variation Patterns

**Figure 10:**
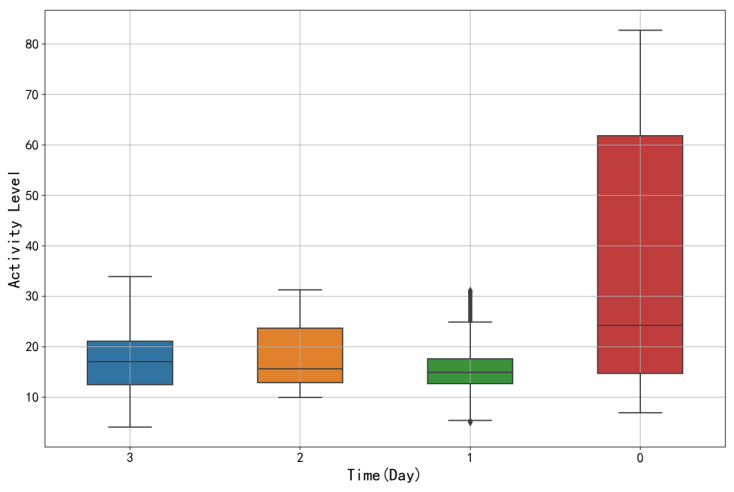
Box plot of sow_16 activity distribution relative to farrowing onset. Time intervals on the x-axis: 0 (0–24 h before onset), 1 (24–48 h before onset), 2 (48–72 h before onset), and 3 (72–96 h before onset).

In this study, we conducted an in-depth analysis of the activity variation patterns of sow_16 to explore the feasibility of predicting farrowing time based on changes in sow activity. Figure 10 presents a box plot showing the daily activity distribution of sow_16, while Table 4 lists the specific values of the box plot elements (with “0” representing the time from farrowing onset to 24 h before farrowing, and so on). Data analysis indicates that between 3 and 1 day prior to farrowing, the activity level of sow_16 remained notably stable. During this period, the mean, minimum, maximum, and median (Q2) values of activity showed small fluctuations, with the interquartile range (IQR) between 4.88 and 10.78 and the standard deviation ranging from 6.1 to 6.93, indicating low dispersion in the activity distribution. This stability reflects the sow’s physiological state and behavioral patterns during this critical period, suggesting that as farrowing approaches, the sow maintains a relatively constant level of activity by regulating its metabolic and physiological processes.

However, on the day of farrowing, the mean, minimum, maximum, and median values of activity significantly increased. At this point, the IQR reached 37.57, and the standard deviation was 26.26, indicating a much larger variability. These changes are likely associated with uterine contractions, fluctuations in hormone levels, and the onset of nesting behavior. The analysis suggests that the variation in sow activity prior to farrowing is closely linked to farrowing events, and these changes provide valuable insights for further research into the physiological state of sows during late pregnancy and for predicting farrowing times.

**Figure 11:**
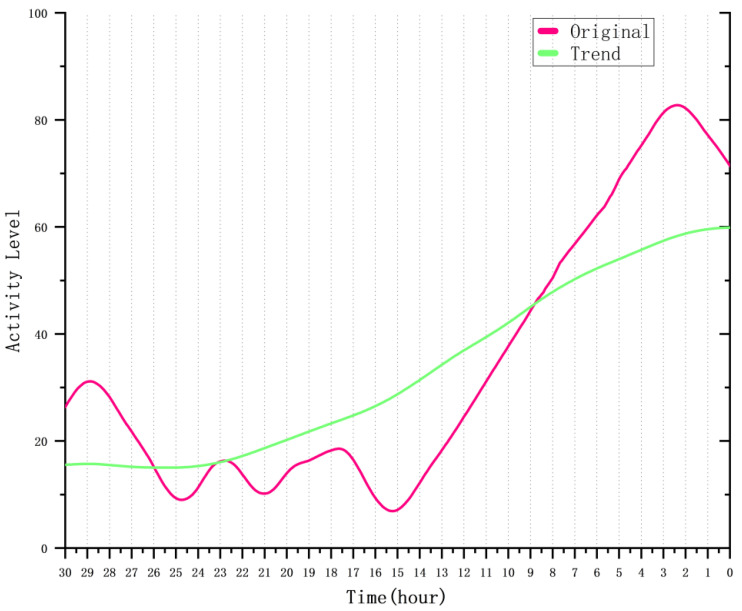
Activity variation in sow_16 before farrowing: original and trend data.

To further investigate the activity variation in sow_16 in the 30 h prior to farrowing, we considered the impact of diurnal rhythms on the sow’s activity. We applied seasonal and trend decomposition using Loess (STL) to separate and remove these cyclical fluctuations. The STL method decomposes the time series into trend, seasonality, and residual components [46]. In this analysis, we retained the trend component. Figure 11 shows the activity variation from 30 h to 0 h before farrowing, with the red line representing the original activity data and the green line representing the trend component. The results indicate that from 30 h to 15 h before farrowing, the sow’s activity followed a clear diurnal rhythm. However, from 15 h to 2 h before farrowing, there was a significant upward trend in activity. For the trend component, the sow’s activity began to increase 24 h prior to farrowing.

In conclusion, the activity variation pattern of sow_16 provides strong support for predicting the onset of farrowing based on activity data. By analyzing the stability and trend of the activity data, particularly the significant changes as farrowing approaches, we can provide effective input features for CLA-PTNet, thereby improving the accuracy and reliability of farrowing predictions.

### 4.5. Predictive Performance of Sow Farrowing Onset Estimation

#### 4.5.1. Analysis of Farrowing Onset Prediction Accuracy

In this study, we used the trained CLA-PTNet model to predict the farrowing onset times for four sows (sow_08, sow_09, sow_17, and sow_20) in the test set, and we conducted an in-depth analysis of the prediction results. The prediction time window ranged from 96 h before farrowing to the actual farrowing time, with a focus on the period from 30 h to 0 h before farrowing. The detailed analysis is presented in Table 5. During the 30 to 24 h before farrowing, the MAE and RMSE values were relatively high, and the R^2^ value was negative, indicating low prediction accuracy during this time period. The significant deviation between the predicted and actual values suggests that the sow’s activity levels had not yet shown significant changes, making it difficult for the model to capture sufficient predictive signals.

However, as the farrowing time approached, from 24 to 18 h before farrowing, the MAE and RMSE values significantly decreased, and the R^2^ value improved substantially, indicating a marked improvement in prediction accuracy. This improvement can be attributed to significant fluctuations in the sow’s activity levels during this period, which became a key feature enabling the model to predict farrowing time more accurately.

Between 18 and 0 h before farrowing, the MAE and RMSE values further decreased, and the R^2^ value continued to increase, showing that CLA-PTNet achieved even higher prediction accuracy as farrowing neared. This enhancement is likely related to the clearer and more pronounced changes in the sow’s activity as the farrowing event approached. To further validate this hypothesis, we analyzed the activity trend components of sow_08, sow_09, sow_17, and sow_20 in the 30 h before farrowing, along with the corresponding farrowing onset time predictions.

**Figure 12:**
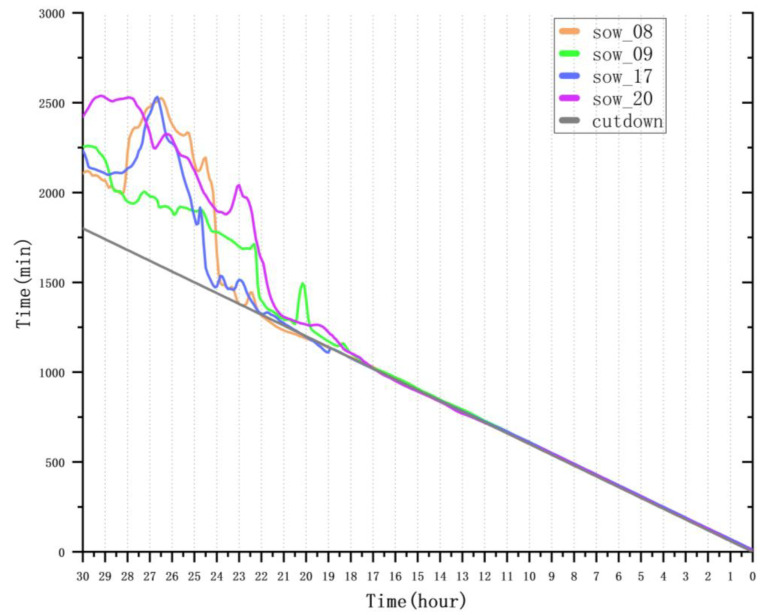
Comparison of predicted and actual values.

**Figure 13:**
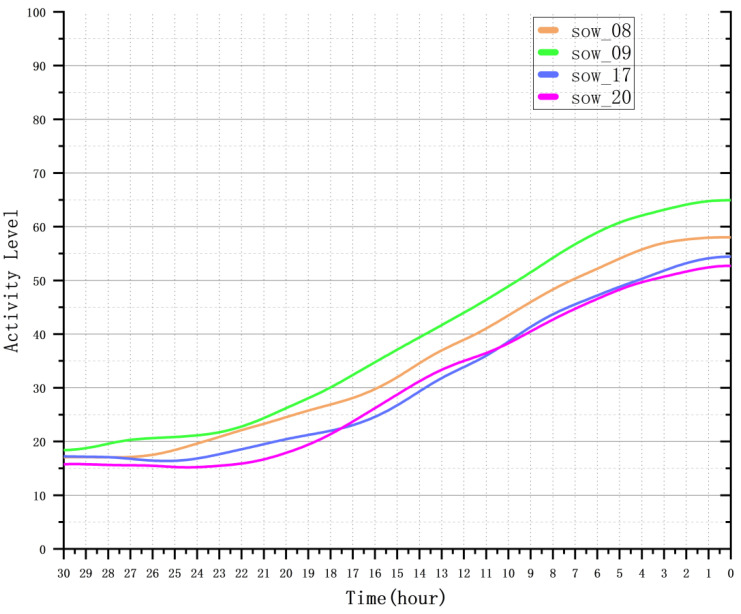
Activity trend component.

As shown in Figure 12 and Figure 13, for sow_08, the prediction error began to decrease around 25 h before farrowing, with the rise in the activity trend component becoming more pronounced between 26 and 25 h before farrowing. For sow_09, the prediction error started to decrease more rapidly around 22.5 h before farrowing, with the activity trend component showing a rise between 23 and 22 h before farrowing. Similarly, for sow_17, the prediction error decreased sharply around 25 h before farrowing, with the rise in the activity trend component occurring between 26 and 25 h before farrowing. For sow_20, the prediction error decreased rapidly around 22 h before farrowing, with the activity trend component rising between 22 and 21 h before farrowing. These results indicate that CLA-PTNet effectively captures significant changes in sow activity, thereby improving the accuracy of farrowing predictions.

In conclusion, CLA-PTNet can capture the linear change in remaining time to farrowing based on sow activity variation patterns. The approach to farrowing exhibits relatively stable and predictable characteristics, and the model shows high sensitivity to activity changes during specific time periods. This suggests that CLA-PTNet can leverage these key features to significantly improve the accuracy of farrowing time predictions. Recognizing this activity variation pattern is crucial for early farrowing alerts. The ability to predict farrowing times based on activity data has significant practical value for farmers, as it can optimize farm management, reduce farrowing risks, and improve production efficiency.

#### 4.5.2. Performance Comparison of CLA-PTNet with Other Models

To further evaluate the effectiveness of CLA-PTNet, we compared its performance with two widely used deep learning models: LSTM and CNN-LSTM. The evaluation was conducted on the same test dataset, which consisted of four sows (sow_08, sow_09, sow_17, and sow_20). The prediction performance was specifically assessed for the 18-hour period before farrowing (18–0 h), as this is the critical window for early intervention in farm management. The comparison focused on three key predictive performance metrics: MAE, RMSE, and R^2^, as previously defined. Additionally, the number of model parameters was considered to assess computational complexity. The results of this comparison are summarized in Table 6.

The results indicate that CLA-PTNet significantly outperforms both LSTM and CNN-LSTM in farrowing onset prediction accuracy. The MAE of CLA-PTNet is 5.42 min, which is much lower than that of CNN-LSTM (68.66 min) and LSTM (86.34 min), indicating that CLA-PTNet produces far smaller errors in predicting farrowing onset time. Similarly, CLA-PTNet achieves the lowest RMSE of 5.97 min, while CNN-LSTM and LSTM have much higher RMSE values of 70.36 min and 98.76 min, respectively, showing that CLA-PTNet not only minimizes overall prediction errors but also effectively reduces large deviations. The high R^2^ value of 0.99 further confirms that CLA-PTNet provides a nearly perfect fit between predicted and actual values, whereas CNN-LSTM and LSTM have R^2^ values of 0.96 and 0.94, respectively, indicating weaker predictive capabilities.

The superior performance of CLA-PTNet can be attributed to its structural advantages, which include the integration of convolutional neural networks (CNNs) for feature extraction, long short-term memory (LSTM) networks for capturing long-range dependencies, and the Position-Aware Time-Weighted Self-Attention (PTW-SA) mechanism for adaptive weighting of time-series features. In contrast, the LSTM model, despite being effective for sequence modeling, lacks the ability to differentiate the importance of different time steps, leading to suboptimal prediction accuracy. The CNN-LSTM model, which combines convolutional layers with LSTM, improves feature extraction over standard LSTM but still does not fully address the need to emphasize critical activity changes. By incorporating PTW-SA, CLA-PTNet effectively assigns higher weights to key time steps that are more relevant to farrowing onset, enabling the model to focus on the most critical behavioral changes.

While achieving superior prediction accuracy, CLA-PTNet maintains a reasonable computational footprint. The model contains only 0.76 million parameters, which is slightly higher than LSTM (0.65 M) and CNN-LSTM (0.71 M) but still remains within a practical range for real-time applications.

The results demonstrate that CLA-PTNet achieves superior prediction accuracy and robustness compared to LSTM and CNN-LSTM, particularly in the final 18 h before farrowing, when early intervention is most critical. By leveraging CNN for spatial feature extraction, LSTM for capturing temporal dependencies, and PTW-SA for adaptive attention weighting, CLA-PTNet effectively models complex activity variations, enabling precise farrowing onset prediction.

#### 4.5.3. Impact of Sliding Window Length on Prediction Performance

To investigate the effect of different sliding window lengths on the prediction performance of sow farrowing onset, we conducted comparative experiments using four different window sizes: 3 h, 6 h, 12 h, and 24 h. The evaluation was performed on the four sows in the test set (sow_08, sow_09, sow_17, and sow_20), focusing on the prediction window from 18 h before farrowing to the onset of farrowing. The results of this comparison are presented in Table 7.

The findings indicate that the choice of sliding window length significantly impacts the model’s predictive accuracy and computational efficiency. When using a 3-hour window, the model yielded an MAE of 8.62 min and an RMSE of 9.37 min, demonstrating relatively high prediction errors. This suggests that a shorter window may not provide sufficient historical activity patterns for the model to accurately capture the sow’s behavioral trends leading up to farrowing. However, the inference time was the lowest at 12.24 ms, indicating a faster prediction process.

With a 6-hour sliding window, the model achieved the best overall performance, with the lowest MAE (5.42 min) and RMSE (5.97 min), while maintaining a reasonable inference time of 22.75 ms. This balance indicates that a 6-hour window provides an optimal trade-off between capturing relevant temporal dependencies and computational efficiency, allowing the model to detect key activity fluctuations more effectively.

As the sliding window length increased to 12 h and 24 h, the MAE and RMSE slightly increased. Specifically, the 12-hour window resulted in an MAE of 5.86 min and an RMSE of 6.35 min, while the 24-hour window further increased the MAE and RMSE to 6.18 min and 7.01 min, respectively. These results suggest that longer sliding windows may introduce excessive historical data, potentially diluting the importance of recent activity variations and reducing the model’s sensitivity to imminent farrowing-related behavioral changes. Additionally, the inference time for the 12-hour and 24-hour windows increased significantly to 44.23 ms and 81.07 ms, respectively, making them less practical for real-time applications.

Overall, the experimental results confirm that a 6-hour sliding window provides the most effective balance between prediction accuracy and computational efficiency. It captures sufficient historical behavioral patterns without overloading the model with excessive past data, ensuring that the prediction remains both accurate and computationally feasible for practical deployment in pig farming environments.

## 5. Discussion

The comparison between our proposed method and previous studies highlights the improvements in prediction accuracy, robustness, and practical applicability achieved by CLA-PTNet. Existing approaches for farrowing prediction have primarily relied on physiological sensors, behavioral tracking, and computer vision-based methods, each with varying degrees of effectiveness.

Sensor-based methods, such as accelerometers combined with CUSUM charts, have been effective in detecting increased activity prior to farrowing. One study reported an average prediction lead time of 13 ± 4.8 h, with high sensitivity (96.7%) but a relatively large standard deviation (4.8 h), limiting its reliability for precise farrowing management [38]. Similarly, a Hidden Phase-type Markov Model (HPMM) using water consumption and activity monitoring provided true warnings in 97% of cases, with an average warning duration of 11.5 h before farrowing. While effective, these methods rely on invasive sensor-based monitoring, which may introduce stress to sows and management challenges [36].

Computer vision-based approaches have also been investigated, such as YOLOX combined with Kalman filtering, which generated first-stage alarms approximately 12 h before farrowing, yet failed to issue alarms for 45% of sows, demonstrating limitations in detection consistency [40]. Another study using YOLOv5 for posture frequency analysis successfully provided early warnings 5 h before farrowing, achieving a mean prediction error of 1.02 h and precision of 93.5% [16]. While these methods have shown promising results, their reliance on predefined behavioral thresholds limits their adaptability across different farming conditions.

Compared to these approaches, CLA-PTNet, integrating CNN, LSTM, and PTW-SA, demonstrates superior predictive accuracy and adaptability. Unlike discrete alarm-based methods, it continuously estimates the remaining time until farrowing, allowing for precise, real-time monitoring. In the final 18 h before farrowing, CLA-PTNet achieves a mean absolute error (MAE) of just 5.42 min, significantly reducing prediction errors compared to previous methods. This accuracy provides farm personnel with reliable insights for optimized management.

To evaluate the economic feasibility of CLA-PTNet, we conducted a ROI analysis, focusing on its potential to reduce stillbirth losses and labor costs. The ROI is calculated as follows:

(16) $$ROI=\frac{Net\;Benefit\;from\;prediction\;System-Cost\;of\;Implementation}{Cost\;of\;Implementation}\times 100\%$$

The net benefit primarily comes from two sources: reduction in stillbirth losses and labor cost savings. The reduction in stillbirth losses is computed as the number of piglets saved per sow, multiplied by the average market value per piglet:

(17) $$B_{p}=∆P\times V_{p}$$

where ∆P represents the decrease in stillbirths per sow, and $V_{p}$ is the market price of each piglet. Similarly, labor cost savings are derived from the reduction in monitoring time, multiplied by the hourly wage of farm personnel:

(18) $$B_{l}=∆L\times W_{l}V_{p}$$

where ∆L is the reduction in labor hours per sow, and $W_{l}$ is the hourly wage. The total implementation cost includes both equipment costs for cameras and computing hardware, and operational costs for data processing and maintenance:

(19) $$C_{total}=C_{e}\times C_{o}$$

By applying estimated values based on industry data, the analysis demonstrates that the CLA-PTNet system offers a positive ROI, validating its economic feasibility.

While CLA-PTNet was initially evaluated on high-performance hardware, its architecture was specifically designed for computational efficiency, making it suitable for resource-constrained farm environments. The model operates on structured time-series activity values, rather than raw video data, significantly reducing computational load. With only 0.76 million parameters, CLA-PTNet remains lightweight and feasible for deployment on edge computing devices, such as mid-range AI processors, edge GPUs, or industrial farm servers.

The primary computational bottleneck in our system lies in optical flow estimation. While RAFT provides highly accurate motion estimation, its iterative refinement mechanism demands substantial computational resources, making real-time inference on low-power devices challenging. To address this, future work will explore alternative optical flow models optimized for embedded AI inference, enabling real-time execution in farm environments. Additionally, frame downsampling, model quantization, and hybrid edge-cloud architecture could further reduce computational overhead while maintaining prediction accuracy.

The practical feasibility of deploying CLA-PTNet depends on optimizing both the prediction model and motion estimation process. While CLA-PTNet itself is well-suited for real-time farm deployment, the computational burden of optical flow estimation must be addressed. Future research will focus on reducing this computational cost, ensuring that the system remains scalable, cost-effective, and viable for large-scale farm adoption. These improvements will enhance on-farm automation capabilities, allowing for non-invasive, real-time farrowing prediction without requiring high-end computing infrastructure.

To further extend the applicability of CLA-PTNet, future research should explore its potential for distinguishing physiological farrowing from pathological conditions that require intervention. While our current study focuses on accurately predicting farrowing onset, integrating additional physiological markers, such as body temperature, heart rate, and vocalization patterns, could enhance the model’s ability to detect abnormal labor events. This would allow for early identification of complications, enabling timely veterinary intervention to reduce the risk of stillbirths and improve sow welfare. Additionally, incorporating multi-modal data sources, such as thermal imaging and real-time monitoring of maternal behavior, could provide a more comprehensive assessment of sow health during farrowing. Future studies should investigate how these additional factors can be combined with activity-based prediction models to enhance the clinical relevance and practical utility of AI-driven farrowing monitoring systems in commercial farming.

## 6. Conclusions

This study proposed a novel method to predict farrowing onset in late-pregnancy sows using optical flow estimation and deep learning techniques. Sow activity was accurately quantified from visible-light videos through the RAFT algorithm, and activity reliability was validated by a strong correlation across sows (average Pearson correlation coefficient of 0.819). Notably, sow_16 displayed distinct activity variations before farrowing, with significant increases occurring 24 h prior to the event.

For farrowing onset prediction, the proposed CLA-PTNet model was able to accurately predict the farrowing time, especially as the event approached. The model demonstrated significantly improved accuracy, particularly in capturing critical time points. During the 18 to 0 h before farrowing, the average MAE, RMSE, and R^2^ values across four sows in the test set were 5.42 min, 5.97 min, and 0.99, respectively. This result indicates that CLA-PTNet can accurately predict farrowing onset time up to 18 h before the event.

Although the model’s performance is promising, further validation across larger populations and different farming environments is essential to ensure broader applicability and reliability. Overall, this method offers valuable potential for enhancing sow management and improving piglet welfare in commercial pig farming.

## Figures and Tables

**Table 1:** Distribution of the sow-activity time-series dataset.

Dataset	Number of Sows
Training set	12
Validation set	4
Test set	4

**Table 2:** Performance comparison of different optical flow estimation models on Sintel and KITTI datasets. Abbreviations: EPE (End-Point Error, average pixel distance; lower is better); Fl-all (% of pixels with error > 3 px or 5% flow magnitude; lower is better). Models: FlowNet2 (Flow Network v2), GMA (Global Motion Aggregation), MaskFlowNet, PWC-Net (Pyramid, Warping, and Cost Volume Network), and RAFT (Recurrent All-Pairs Field Transforms). Datasets: Sintel Clean (synthetic, noise-free), Sintel Final (synthetic with motion blur/lighting effects), and KITTI2015 (real-world driving scenes).

Model	Sintel Clean EPE (Pixels)	Sintel Final EPE (Pixels)	KITTI2015 EPE (Pixels)	KITTI2015 Fl-All	Parameter (M)
FlowNet2	1.96	3.69	8.23	28.28%	162
GMA	1.48	2.93	6.73	19.17%	5.9
MaskFlowNet	2.30	3.73	9.36	29.70%	42
PWC-Net	2.26	3.79	9.49	29.85%	9.3
RAFT	1.38	2.79	4.95	16.23%	5.3

**Table 3:** AACC between each sow and the other 19 sows. AACC (average activity correlation coefficient).

Sow ID	AACC	Sow ID	AACC	Sow ID	AACC	Sow ID	AACC
sow_01	0.804	sow_06	0.783	sow_11	0.817	sow_16	0.870
sow_02	0.810	sow_16	0.793	sow_12	0.850	sow_17	0.864
sow_03	0.813	sow_08	0.832	sow_13	0.854	sow_18	0.847
sow_04	0.853	sow_09	0.784	sow_14	0.826	sow_19	0.668
sow_05	0.835	sow_10	0.818	sow_15	0.852	sow_20	0.811

**Table 4:** Statistical indicators of daily activity distribution for sow_16.

Date	Mean	Min	Max	Q1	Q2	Q3	SD
3	17.21	4.09	33.90	12.49	17.04	21.07	6.93
2	18.31	9.96	31.27	12.90	15.60	23.68	6.70
1	15.75	5.00	31.15	12.70	14.92	17.58	6.10
0	36.83	6.89	82.75	14.70	24.26	61.83	26.26

**Table 5:** Prediction results for different time periods in the 30 hours before farrowing.

Sow ID	Hours Before Farrow	MAE (min)	RMSE (min)	R^2^
sow_08	30–24	609.82	651.47	−38.31
	24–18	22.74	34.74	0.89
	18–12	3.48	4.34	0.99
	12–6	4.08	4.52	0.99
	6–0	2.53	2.98	0.99
sow_09	30–24	367.70	372.49	−11.85
	24–18	144.99	196.25	−2.56
	18–12	9.98	10.68	0.98
	12–6	7.72	8.17	0.99
	6–0	6.36	6.54	0.99
sow_17	30–24	489.20	538.64	−25.87
	24–18	31.53	48.52	0.78
	18–12	4.61	5.18	0.99
	12–6	5.62	5.66	0.99
	6–0	4.97	5.13	0.99
sow_20	30–24	708.01	716.08	−46.49
	24–18	222.45	310.63	−7.94
	18–12	8.39	10.21	0.99
	12–6	4.06	4.77	0.99
	6–0	3.35	3.49	0.99

**Table 6:** Performance comparison of different models for farrowing onset prediction (18–0 h before farrowing).

Model	MAE (min)	RMSE (min)	R^2^	Parameter (M)
LSTM	86.34	98.76	0.94	0.65
CNN-LSTM	68.66	70.36	0.96	0.71
CLA-PTNet	5.42	5.97	0.99	0.76

**Table 7:** Impact of sliding window length on prediction performance.

Sliding Windows (h)	MAE (min)	RMSE (min)	Inference Time (ms)
3	8.62	9.37	12.24
6	5.42	5.97	22.75
12	5.86	6.35	44.23
24	6.18	7.01	81.07

## Data Availability

Data are contained within the article.
